# Supplementary material for: Interrogating the recognition landscape of a conserved HIV-specific TCR reveals distinct bacterial peptide cross-reactivity
Source: eLife. 2020 Jul 27;9:e58128. doi: 10.7554/eLife.58128 (PMC7384859; doi:10.7554/eLife.58128)
Supplement: Supplementary file 1. — Table 1: Round 3 of the AGA-1 TCR yeast display selection results were used to identify sequence related peptides from the non-redundant ('nr') database. Listed are the prediction results for KF11-related peptides derived from the gag HIV protein. GI identification and blast scores are included. Table 2: AGA-1 TCR yeast display selection results from Round 3 screens were used to identify sequence related peptides from the non-redundant ('nr') database. Listed are the prediction results of sequence-related, non-KF11 peptides. GI identification, blast scores, % identity to the KF11 peptide and to 'nr' database mined peptide hits are included. Table 3: TCR alpha chain amino acid sequence identity of clone 1.2 and the AGA1 (clone 1.1) TCR. Clones 1.1 and 1.2 both utilize the V alpha 5 (AV5) chain segments, encode identical CDR3 motifs and carry one amino acid sequences difference that maps to the J alpha (AJ) region (underlined). This residue is outside the TCR:peptide-MHC binding interface described previously for the AGA1 TCR-B*57:03-KF11 co-complex (red) (Stewart-Jones et al., 2012). AGA1 TCR clone 1.1 and the related clone 1.2 are 100% sequence identical across the CDR3 and J regions of their V beta19 (BV19) TCR chain sequences (CASTGSYGYTFGSGTRLTVT) (Stewart-Jones et al., 2012). The TCR V region, CDR3 and J region boundaries as defined by ImMunoGeneTics (IMGT), http://www.imgt.org/IMGTrepertoire/. Table 4: Peptide binding strength hierarchy ranked on the basis of UV exchange HLA-B*57:01 peptide binding ELISA data. The index KF11 epitope and the HdH peptides are ranked in descending order according to their peptide binding strength as determined using the UV exchange peptide binding assay. High, medium and low binders are color-coded from dark to light shades of grey, respectively. Amino acids differences specific to low/medium binders outside the p4-p6 TCR recognition interface are highlighted (bold squared). p1 = peptide position 1, etc. [file elife-58128-supp1.pptx]

## Slide 1
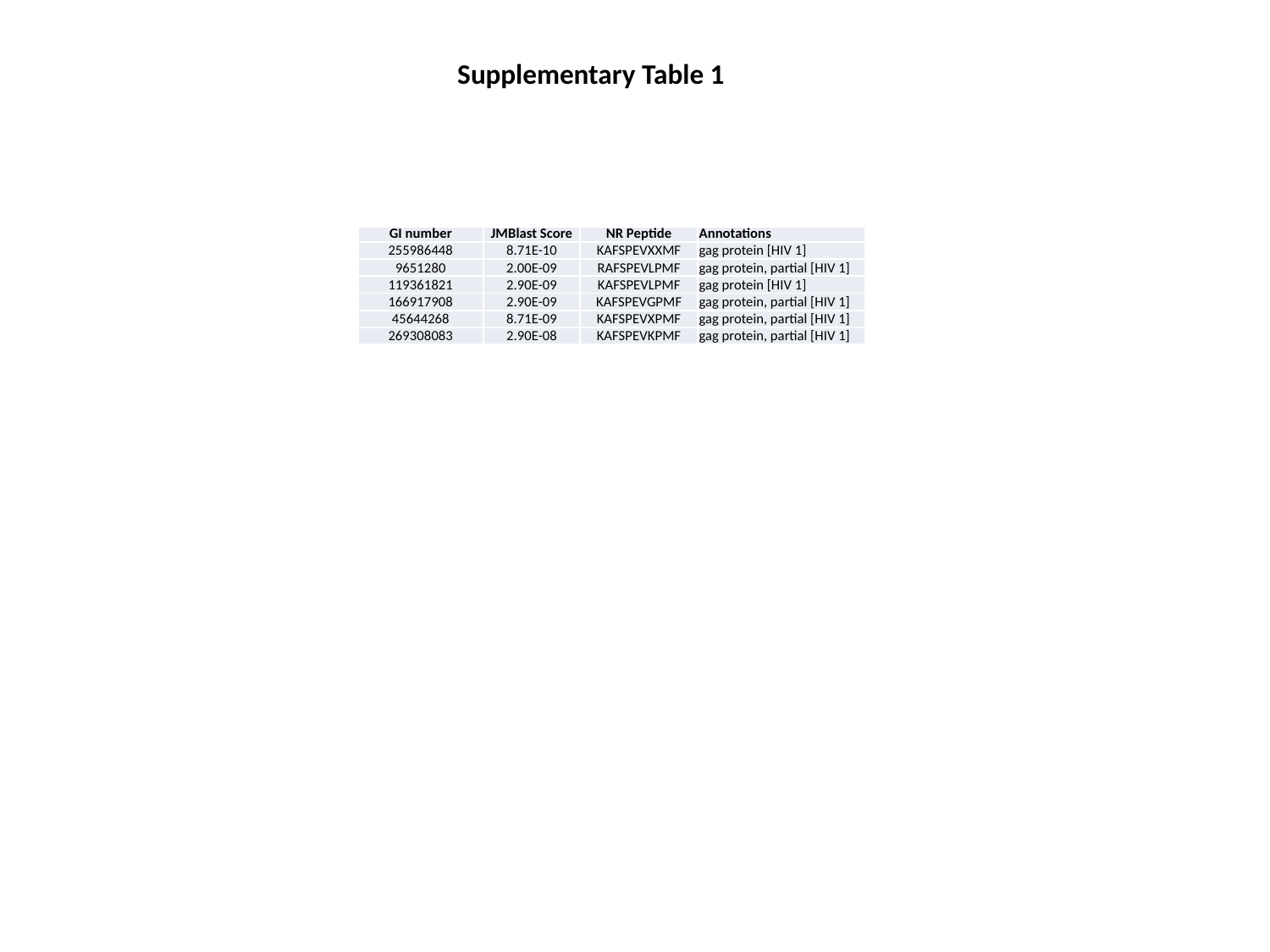

# Supplementary Table 1
| GI number | JMBlast Score | NR Peptide | Annotations |
| --- | --- | --- | --- |
| 255986448 | 8.71E-10 | KAFSPEVXXMF | gag protein [HIV 1] |
| 9651280 | 2.00E-09 | RAFSPEVLPMF | gag protein, partial [HIV 1] |
| 119361821 | 2.90E-09 | KAFSPEVLPMF | gag protein [HIV 1] |
| 166917908 | 2.90E-09 | KAFSPEVGPMF | gag protein, partial [HIV 1] |
| 45644268 | 8.71E-09 | KAFSPEVXPMF | gag protein, partial [HIV 1] |
| 269308083 | 2.90E-08 | KAFSPEVKPMF | gag protein, partial [HIV 1] |

## Slide 2
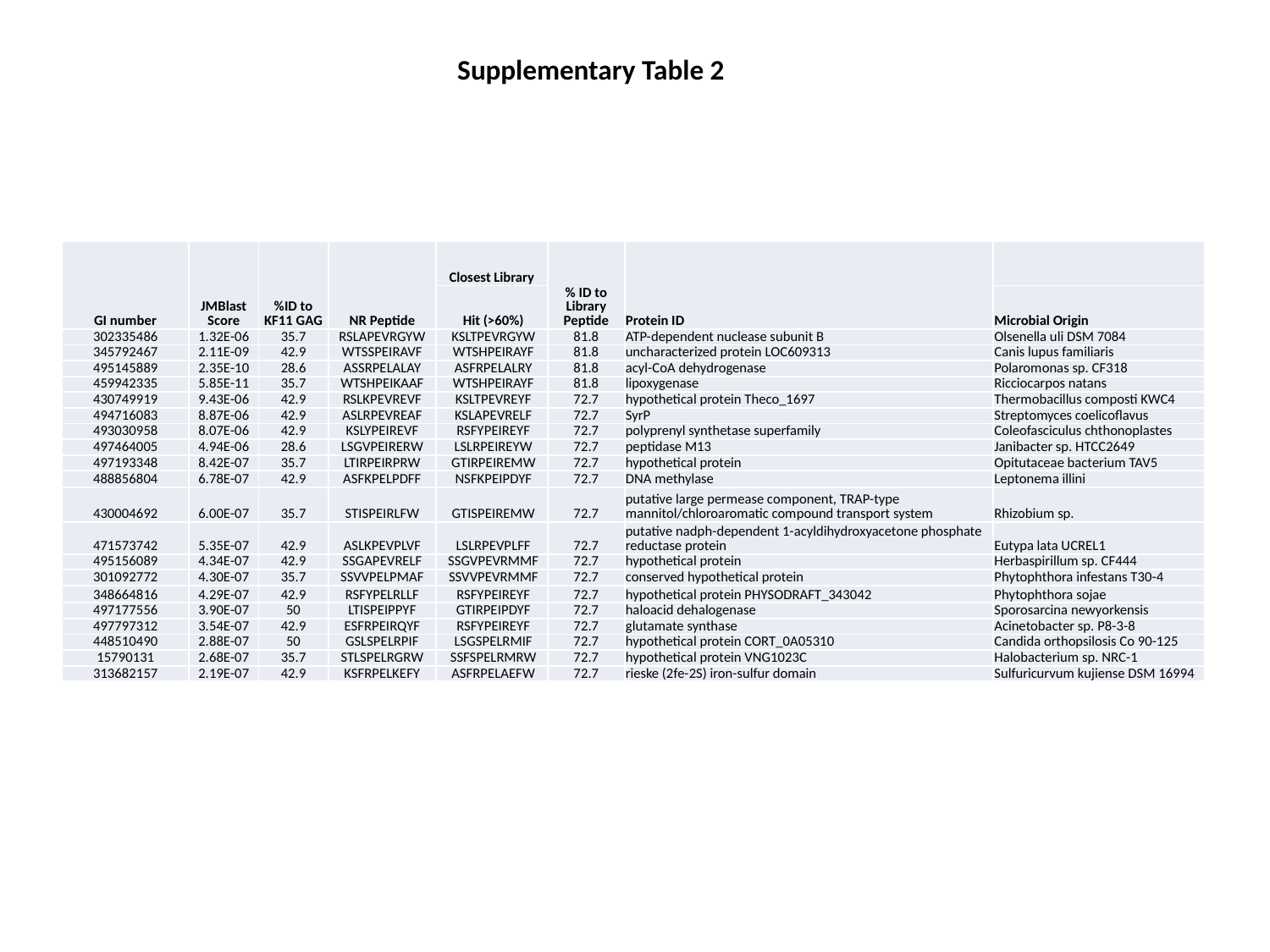

# Supplementary Table 2
| GI number | JMBlast Score | %ID to KF11 GAG | NR Peptide | Closest Library | % ID to Library Peptide | Protein ID | |
| --- | --- | --- | --- | --- | --- | --- | --- |
| | | | | Hit (>60%) | | | Microbial Origin |
| 302335486 | 1.32E-06 | 35.7 | RSLAPEVRGYW | KSLTPEVRGYW | 81.8 | ATP-dependent nuclease subunit B | Olsenella uli DSM 7084 |
| 345792467 | 2.11E-09 | 42.9 | WTSSPEIRAVF | WTSHPEIRAYF | 81.8 | uncharacterized protein LOC609313 | Canis lupus familiaris |
| 495145889 | 2.35E-10 | 28.6 | ASSRPELALAY | ASFRPELALRY | 81.8 | acyl-CoA dehydrogenase | Polaromonas sp. CF318 |
| 459942335 | 5.85E-11 | 35.7 | WTSHPEIKAAF | WTSHPEIRAYF | 81.8 | lipoxygenase | Ricciocarpos natans |
| 430749919 | 9.43E-06 | 42.9 | RSLKPEVREVF | KSLTPEVREYF | 72.7 | hypothetical protein Theco\_1697 | Thermobacillus composti KWC4 |
| 494716083 | 8.87E-06 | 42.9 | ASLRPEVREAF | KSLAPEVRELF | 72.7 | SyrP | Streptomyces coelicoflavus |
| 493030958 | 8.07E-06 | 42.9 | KSLYPEIREVF | RSFYPEIREYF | 72.7 | polyprenyl synthetase superfamily | Coleofasciculus chthonoplastes |
| 497464005 | 4.94E-06 | 28.6 | LSGVPEIRERW | LSLRPEIREYW | 72.7 | peptidase M13 | Janibacter sp. HTCC2649 |
| 497193348 | 8.42E-07 | 35.7 | LTIRPEIRPRW | GTIRPEIREMW | 72.7 | hypothetical protein | Opitutaceae bacterium TAV5 |
| 488856804 | 6.78E-07 | 42.9 | ASFKPELPDFF | NSFKPEIPDYF | 72.7 | DNA methylase | Leptonema illini |
| 430004692 | 6.00E-07 | 35.7 | STISPEIRLFW | GTISPEIREMW | 72.7 | putative large permease component, TRAP-type mannitol/chloroaromatic compound transport system | Rhizobium sp. |
| 471573742 | 5.35E-07 | 42.9 | ASLKPEVPLVF | LSLRPEVPLFF | 72.7 | putative nadph-dependent 1-acyldihydroxyacetone phosphate reductase protein | Eutypa lata UCREL1 |
| 495156089 | 4.34E-07 | 42.9 | SSGAPEVRELF | SSGVPEVRMMF | 72.7 | hypothetical protein | Herbaspirillum sp. CF444 |
| 301092772 | 4.30E-07 | 35.7 | SSVVPELPMAF | SSVVPEVRMMF | 72.7 | conserved hypothetical protein | Phytophthora infestans T30-4 |
| 348664816 | 4.29E-07 | 42.9 | RSFYPELRLLF | RSFYPEIREYF | 72.7 | hypothetical protein PHYSODRAFT\_343042 | Phytophthora sojae |
| 497177556 | 3.90E-07 | 50 | LTISPEIPPYF | GTIRPEIPDYF | 72.7 | haloacid dehalogenase | Sporosarcina newyorkensis |
| 497797312 | 3.54E-07 | 42.9 | ESFRPEIRQYF | RSFYPEIREYF | 72.7 | glutamate synthase | Acinetobacter sp. P8-3-8 |
| 448510490 | 2.88E-07 | 50 | GSLSPELRPIF | LSGSPELRMIF | 72.7 | hypothetical protein CORT\_0A05310 | Candida orthopsilosis Co 90-125 |
| 15790131 | 2.68E-07 | 35.7 | STLSPELRGRW | SSFSPELRMRW | 72.7 | hypothetical protein VNG1023C | Halobacterium sp. NRC-1 |
| 313682157 | 2.19E-07 | 42.9 | KSFRPELKEFY | ASFRPELAEFW | 72.7 | rieske (2fe-2S) iron-sulfur domain | Sulfuricurvum kujiense DSM 16994 |

## Slide 3
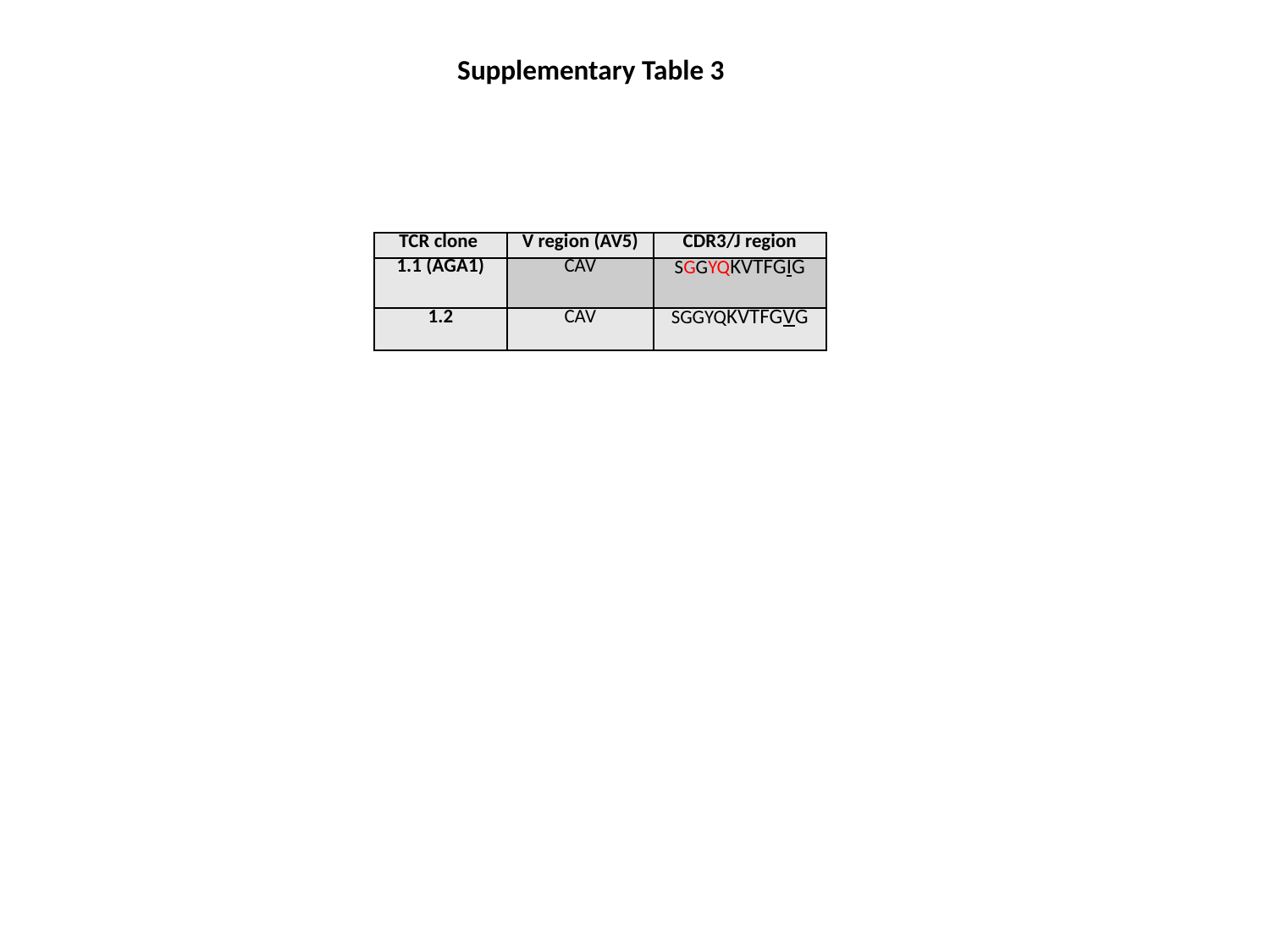

# Supplementary Table 3
| TCR clone | V region (AV5) | CDR3/J region |
| --- | --- | --- |
| 1.1 (AGA1) | CAV | SGGYQKVTFGIG |
| 1.2 | CAV | SGGYQKVTFGVG |

## Slide 4
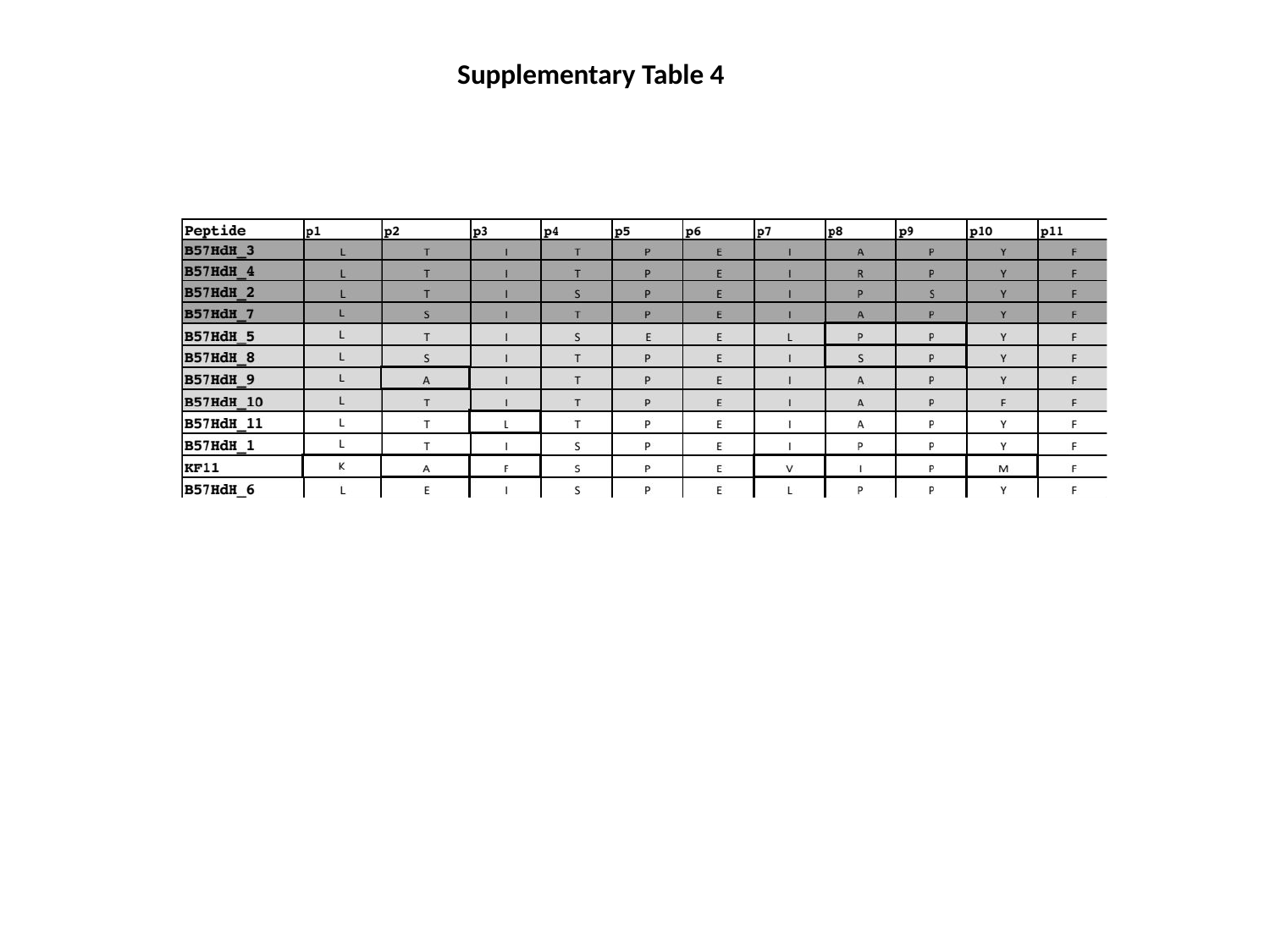

# Supplementary Table 4
